# Surgical management strategies and postoperative outcomes of choledochal cysts in children: the role of preoperative radiological assessment in a retrospective observational study

**DOI:** 10.3389/fped.2026.1880951

**Published:** 2026-08-24

**Authors:** Qi Wang, Peng Cai, Zhenwei Zhu, Jianlei Chen, Haowei Zhao, Menglei Zhu, Xiaogang Zhou, Yuliang Jiang, Chengpeng Shi, Jie Zhu

**Affiliations:** Department of General Surgery, Children’s Hospital of Soochow University, Suzhou, Jiangsu, China

**Keywords:** choledochal cysts, pancreaticobiliary maljunction, prognostic analysis, radiological assessment, Todani classification

## Abstract

**Background:**

Choledochal cysts (CCC) are common congenital biliary malformations in children. Early diagnosis and standardized surgery are critical for prognosis. Preoperative imaging is essential for classification and surgical planning, but the value of different modalities remains unclear.

**Purpose:**

This study aimed to investigate the value of preoperative imaging in guiding surgical strategies for pediatric CCC, analyze its prognostic predictive efficiency, and explore related risk factors to provide evidence for individualized clinical management.

**Methods:**

A retrospective study enrolled 104 children with choledochal cysts surgically treated from January 2022 to December 2024. All diagnoses were confirmed by preoperative imaging, intraoperative exploration and postoperative pathology. Cysts were classified by the Todani system. Preoperative imaging included abdominal ultrasound (US), computed tomography (CT), magnetic resonance imaging/magnetic resonance cholangiopancreatography (MRI/MRCP) and intraoperative cholangiography (IOC). Key radiological features were collected. We analyzed the correlation between imaging features and surgical strategy. Primary outcomes were early postoperative complications and mid-term liver function abnormalities; secondary outcomes included operative time, blood loss, hospital stay and reoperation rate. Univariate and multivariate Logistic regression identified independent risk factors. A combined predictive model was constructed, and ROC curves evaluated its predictive performance (95% CI).

**Results:**

The most common types were Todani type I (65.4%) and type IVa (24.0%). Magnetic resonance cholangiopancreatography achieved a classification accuracy of 96.2% and exhibited higher detection rates of pancreaticobiliary maljunction and intrahepatic bile duct dilatation than ultrasound and computed tomography (*P* < 0.05). Cyst resection with Roux-en-Y hepaticojejunostomy was the predominant procedure (81.7%). The overall early postoperative complication rate was 16.3% and incomplete preoperative radiological assessment was an independent risk factor. The combined model of ultrasound and magnetic resonance cholangiopancreatography yielded an AUC of 0.872.

**Conclusion:**

Preoperative imaging is essential for pediatric choledochal cyst management, and the combination of ultrasound and MRCP enables precise surgical planning and better prognosis.

## 1 Introduction

Choledochal cysts (CCC), also known as congenital biliary dilatation (CBD), are common congenital biliary malformations in pediatric surgery, characterized by cystic or fusiform dilatation of the common bile duct, often complicated by pancreaticobiliary maljunction (PBM), bile duct stenosis and biliary stones (1, 2). The incidence is significantly higher in Asia than in Western countries, with a female predominance of approximately 1:3–1:4 (3–5). The pathogenesis of pediatric CCC remains incompletely understood, with PBM widely recognized as the core mechanism: the premature union of the pancreatic and bile ducts outside the duodenal wall leads to pancreatic juice reflux into the bile duct, triggering mucosal inflammation and injury, and subsequent cyst formation (2). In addition, congenital defects in the bile duct wall and biliary obstruction may also contribute to disease progression (6).

Untreated pediatric CCC can progress to severe complications such as biliary perforation, cholangitis and pancreatitis (7, 8), and even increase the risk of cholangiocarcinoma in adulthood, making early diagnosis and standardized treatment critical for improving prognosis (3). Surgery is the only curative treatment for pediatric CCC, with core principles of complete cyst resection, relief of biliary obstruction, reconstruction of biliary drainage and prevention of pancreatic reflux (9). Currently, cyst resection combined with biliary enteric anastomosis is the standard procedure, but surgical strategies must be tailored to cyst type, anatomical variations and associated lesions (10, 11).

Accurate preoperative evaluation of cyst morphology, extent of involvement, PBM and intrahepatic bile duct disease is essential for formulating optimal surgical plans and minimizing early postoperative complications (12, 13). With advances in imaging technology, modalities including US, MRCP and CT have been widely used in the diagnosis of CCC (14–16), yet the value of different imaging combinations in guiding surgical decision-making and predicting postoperative outcomes remains to be systematically evaluated, and the correlation between preoperative radiological features and early postoperative complications has not been fully elucidated.

Therefore, this retrospective study analyzed 104 children with CCC who underwent surgical treatment at our hospital, to explore the role of preoperative radiological assessment in surgical strategy selection, analyze the predictive value of radiological parameters for postoperative outcomes, and identify independent prognostic risk factors, aiming to provide a theoretical basis for standardized diagnosis and individualized surgical management of pediatric CCC.

## 2 Materials and methods

### 2.1 Study design

**Figure 1:**
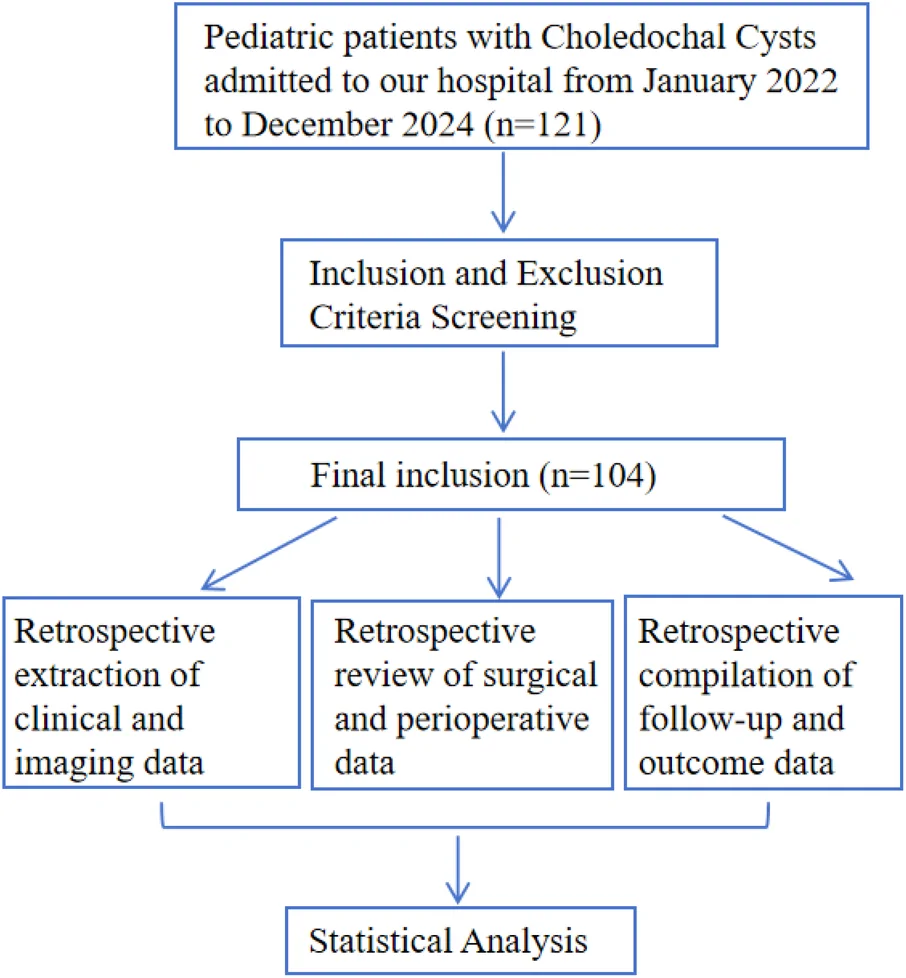
Study flowchart.

This was a single-center retrospective observational study. The study protocol was reviewed and approved by the Medical Ethics Committee of our hospital. Owing to the retrospective design, written informed consent was waived by the ethics committee. The study was conducted in strict accordance with the ethical principles outlined in the Declaration of Helsinki, ensuring patient privacy and data security. The study flowchart is shown in Figure 1.

### 2.2 Inclusion and exclusion criteria (1, 17)

Eligible patients were identified through the pediatric medical record system, surgical registry and postoperative follow-up database. Inclusion and exclusion criteria were defined as follows:

Inclusion criteria: (1) Age ≤12 years, with a confirmed diagnosis of congenital choledochal cyst based on preoperative imaging, intraoperative exploration and postoperative histopathology, in accordance with relevant guidelines and literature; (2) Underwent radical surgical treatment for choledochal cyst at our pediatric surgery department, without palliative procedures such as simple drainage or stoma creation; (3) Complete preoperative imaging data, surgical records, postoperative pathology reports and mid-term follow-up data available, with a minimum valid follow-up duration of 6 months postoperatively for outcome evaluation.
Exclusion criteria: (1) Complicated with other severe congenital malformations (e.g., congenital heart disease, esophageal atresia, chromosomal abnormalities) that may affect postoperative recovery and outcome assessment; (2) Received only palliative surgery due to critical illness, without radical cyst resection; (3) Incomplete clinical records, preoperative imaging data, surgical notes or follow-up data with missing key information, logical errors or tampering, preventing extraction of core study indicators; (4) Previous history of biliary surgery or major abdominal trauma, leading to anatomical distortion or severe abdominal adhesions that interfere with surgical decision-making and outcome evaluation; (5) Lost to follow-up postoperatively, resulting in inability to assess mid-term outcomes.

### 2.3 Preoperative radiological assessment

All imaging studies were reviewed independently by two pediatric radiologists with ≥5 years of experience, with discrepancies resolved by a third senior radiologist. Data were archived in the picture archiving and communication system (PACS). All imaging examinations and reports strictly followed the institutional standardized pediatric biliary system structured reporting template, which uniformly regulated the extraction of core radiological indicators to ensure consistent and accurate data collection and reduce subjective bias.

Strict definition of incomplete preoperative radiological assessment: Patients who failed to complete the standardized US + MRI/MRCP combined examination protocol before surgery, including simple US examination only, unevaluable key anatomical information (unclear PBM status, undetermined intrahepatic duct involvement scope) due to incomplete MRCP imaging or imaging artifacts, resulting in inability to support accurate surgical planning. Complete assessment was defined as completion of both standardized US and qualified 3.0 T MRCP examinations with clear diagnostic imaging data.

Standardized MRI/MRCP imaging protocol and pediatric sedation management: All preoperative MRI/MRCP examinations were performed on a 3.0 T superconducting magnetic resonance scanner (Siemens, Germany). Core scanning sequences included 3D respiratory-triggered thin-layer MRCP sequences and 2D thick-slab MRCP static sequences, which optimized the display of fine biliary anatomical structures and reduced respiratory motion artifacts. For children aged <3 years (66.3% of the cohort), unified sedation was implemented: oral chloral hydrate (50 mg/kg) was administered 30 min before scanning, and real-time heart rate and blood oxygen saturation were monitored throughout the examination to ensure stable sedation and high-quality imaging acquisition.

Core radiological parameters extracted included: (1) Cyst classification according to the Todani system; (2) Maximum diameter of the cyst; (3) Presence of PBM; (4) Extent and severity of intrahepatic bile duct dilatation; (5) Cyst wall thickness, presence of necrosis or calcification; (6) Pericystic inflammation and degree of abdominal adhesion; (7) Biliary obstruction and secondary liver parenchymal injury.

### 2.4 Surgical treatment protocol (18)

Surgical team qualification standard: All radical cyst resection procedures in this study were performed by senior attending physicians and above with more than 8 years of specialized pediatric biliary surgery experience. All operations and perioperative management were uniformly implemented by a fixed professional pediatric surgery team with standardized surgical procedures, eliminating confounding bias caused by individual differences in surgeon experience and technical proficiency.

Surgical plans were formulated by a multidisciplinary team consisting of pediatric surgeons, radiologists, anesthesiologists and intensivists, tailored to preoperative radiological findings, Todani cyst type, age, nutritional status and liver/renal function. Specific procedures were selected as follows:

(1) Standard procedure: Complete cyst resection + Roux-en-Y hepaticojejunostomy, indicated for Todani type I cysts. It may also be used for Todani type IVa cysts with mild, localized intrahepatic bile duct dilatation and no irreversible liver parenchymal injury. (2) Intraduodenal cyst resection: Exclusive to Todani type III cysts. (3) Hepatic portoplasty: Indicated for cases with hilar bile duct confluence stenosis and mild, localized intrahepatic bile duct dilatation without irreversible liver injury, mostly in type IVa cysts, and some type I cysts with hilar stenosis. (4) Hepatectomy: Reserved for Todani type IVa cysts with localized intrahepatic bile duct dilatation confined to one lobe/segment and irreversible liver parenchymal injury (atrophy, fibrosis, recurrent infection, stone formation). For Todani type V cysts (isolated multiple intrahepatic bile duct dilatation), localized lesions (one lobe/segment) are treated with lobectomy/segmentectomy, while diffuse lesions require evaluation for liver transplantation. (5) Combined procedures: Used for complex cases, including type IVa cysts with extensive intra- and extrahepatic involvement, multiple intrahepatic strictures/dilatations and liver parenchymal injury; type V cysts with multilobar involvement requiring partial resection and biliary enteric drainage; and complex anatomical variations such as PBM, biliary perforation or hilar vascular anomalies.

### 2.5 Outcome measures and follow-up protocol

#### 2.5.1 Outcome measures

Primary outcomes: Early postoperative complications (occurring within 30 days postoperatively, including biliary fistula, anastomotic stenosis, cholangitis, pancreatitis); mid-term liver function abnormalities (persistent elevation of serum transaminases and bilirubin ≥6 months postoperatively).

Secondary outcomes: Operative time, estimated intraoperative blood loss, postoperative hospital stay, reoperation rate and mid-term survival status.

#### 2.5.2 Follow-up implementation

All patients were enrolled in a dedicated pediatric biliary disease follow-up cohort postoperatively, with scheduled reviews at 1, 3, 6 and 12 months, and optional 24-month recheck. Only follow-up data ≥6 months were included in statistical analysis of prognostic outcomes; 1-month and 3-month routine recheck data were only used for postoperative recovery monitoring and excluded from outcome evaluation. Follow-up included physical examination, liver function tests and abdominal US. Patients with suspected complications underwent additional MRCP to clarify the etiology. All follow-up data were entered into a dedicated database.

### 2.6 Statistical analysis

All data were analyzed retrospectively using SPSS 26.0. Continuous variables were tested for normality using the Kolmogorov–Smirnov test: normally distributed data were expressed as mean ± standard deviation (x¯ ± s), with between-group comparisons using independent samples t-test and multiple-group comparisons using one-way ANOVA (LSD-t correction); non-normally distributed data were expressed as median (interquartile range) [M(P25,P75)], with between-group comparisons using the Mann–Whitney U test and multiple-group comparisons using the Kruskal–Wallis H test. Categorical variables were presented as number (percentage) [*n* (%)], with between-group comparisons using the *χ*^2^ test; corrected *χ*^2^ or Fisher's exact test was used when expected frequencies were small. Early postoperative complications were treated as a binary dependent variable. Potential risk factors with *P* < 0.1 in univariate analysis and clinical confounders were included in the independent variable pool for multivariate Logistic regression (forward LR method) to identify independent risk factors. Variance inflation factor (VIF) was used to test for collinearity (VIF < 5 indicates no collinearity). Continuous variables were categorized according to clinical cut-offs, and categorical variables were coded as dummy variables. Regression coefficients (β), standard errors (SE), odds ratios (OR) and 95%CI were reported.

Construction method of US-MRCP combined predictive model: Based on binary multivariate Logistic regression analysis, three core screened radiological variables were included to construct the early postoperative complication prediction model: US-detected cyst diameter (≤5 cm/ > 5 cm), MRCP-detected PBM (yes/no), and MRCP-detected extensive intrahepatic bile duct dilatation (yes/no). All predictors were coded as binary variables uniformly: 0 represented the absence of the risk factor, and 1 represented the presence of the risk factor. The unstandardized partial regression coefficients of the three variables derived from the multivariate logistic regression were 1.21, 1.35 and 1.18 respectively, with similar magnitude and equivalent independent predictive effects. To facilitate rapid clinical bedside calculation, we converted regression coefficients into simplified integer scores following standardized prediction model construction principles: each variable was assigned 1 point. The model risk score calculation formula is as follows: Risk score = cyst diameter category + PBM + intrahepatic dilatation. The total risk score ranged from 0 to 3 points. The total risk score of each patient was calculated to predict the occurrence probability of early complications. A total of 104 patients’ data were included for model verification, and ROC curves were plotted to calculate AUC, optimal cut-off value, sensitivity, specificity and other predictive indicators for subsequent risk stratification.

Predictive performance was classified according to AUC ranges. Baseline characteristics were compared between follow-up outcome groups, and stratified analysis was performed for radiological risk factors, with the *χ*^2^ test used to compare mid-term prognosis differences. All tests were two-sided, with α=0.05 as the significance level (*P* < 0.05 considered statistically significant).

## 3 Results

### 3.1 Baseline clinical characteristics

A total of 104 children with CCC who underwent surgical treatment at our hospital between January 2022 and December 2024 were included, all confirmed by preoperative imaging, intraoperative exploration and postoperative histopathology. Baseline characteristics are shown in Table 1: 42 males (40.4%) and 62 females (59.6%), with a male-to-female ratio of approximately 1:1.48 (deviating from the global pediatric CCC ratio of 1:3.2–1:4, likely due to single-center selection bias). The median age was 3.2 years (range: 2 months–12 years), with 69 infants (66.3%) ≤3 years old and 35 preschool/school-age children (33.7%).

**Table 1:** Baseline clinical characteristics of included patients.

Indicator	No. of cases (proportion/%)/Median (range)
Gender	
Male	42 (40.4)
Female	62 (59.6)
Age	3.2 years (2 months–12 years)
Todani classification	
Type I	68 (65.4)
Type III	7 (6.7)
Type IVa	25 (24.0)
Type V	4 (3.8)

According to the Todani classification, type I cysts were the most common (65.4%, 68/104), followed by type IVa (24.0%, 25/104), type III (6.7%, 7/104) and type V (3.8%, 4/104). No type II or IVb cysts were identified. Preoperative imaging coverage included: 98 patients (94.2%) underwent combined US and MRI/MRCP, 61 (58.7%) received additional abdominal CT, and all patients underwent intraoperative cholangiography to confirm anatomy.

### 3.2 Diagnostic performance of different preoperative imaging modalities

Taking intraoperative exploration and postoperative pathology as the gold standard, the diagnostic accuracy of US, CT and MRI/MRCP for cyst classification was compared, along with their ability to detect key anatomical features including PBM, intrahepatic bile duct dilatation and pericystic inflammation (Table 2).

**Table 2:** Comparison of diagnostic performance of different preoperative imaging modalities.

Imaging modality	Correctly classified cases (accuracy/%)	PBM detection rate/%	Intrahepatic bile duct dilatation detection rate/%	Pericystic inflammation assessment accuracy/%
Abdominal ultrasound (US)	86 (82.7)	76.5	81.2	72.1
Abdominal CT	82 (78.8)	68.9	74.5	91.8
MRI/MRCP	100 (96.2)	92.3	95.7	86.4
Intraoperative cholangiography (IOC)	104 (100.0)	98.1	99.0	94.2
*χ*^2^ value	21.364	18.729	16.583	12.407
*P* value	<0.001	<0.001	<0.001	0.002

Compared with the US and CT groups, the MRI/MRCP group shows statistically significant differences in diagnostic performance (*P* < 0.05); IOC exhibited significantly higher classification accuracy and detection rates of PBM and intrahepatic duct lesions than all preoperative imaging modalities (all *P* < 0.05).

IOC effectively corrected individual preoperative imaging classification deviations. Among all 104 patients, 4 cases (3.8%) had Todani classification upgraded (preoperative MRCP diagnosed as simple IVa type, IOC confirmed combined multifocal intrahepatic malformation), and 2 cases (1.9%) had classification downgraded. A total of 5 cases (4.8%) adjusted the preoperative surgical scheme based on real-time IOC anatomical findings, including expanded intrahepatic duct exploration and modified biliary anastomosis, avoiding insufficient surgical resection or excessive surgical trauma.

MRI/MRCP achieved the highest classification accuracy of 96.2% (100/104), with only 4 type IVa cysts with extensive intrahepatic dilatation misclassified. The detection rates of PBM and subtle intrahepatic bile duct lesions were 92.3% and 95.7%, respectively, significantly superior to other modalities (*P* < 0.05). As the first-line screening tool, US had a classification accuracy of 82.7% (86/104), with high accuracy in determining cyst size and location, but limited sensitivity for subtle intrahepatic bile duct dilatation and PBM. CT had a classification accuracy of 78.8% (82/104), with advantages in evaluating cyst wall thickening, severe pericystic inflammation/adhesion and adjacent organ compression, providing valuable information for predicting complex surgical risks. The difference in classification accuracy among the three modalities was statistically significant (*χ*^2^ = 21.364, *P* < 0.001).

**Figure 2:**
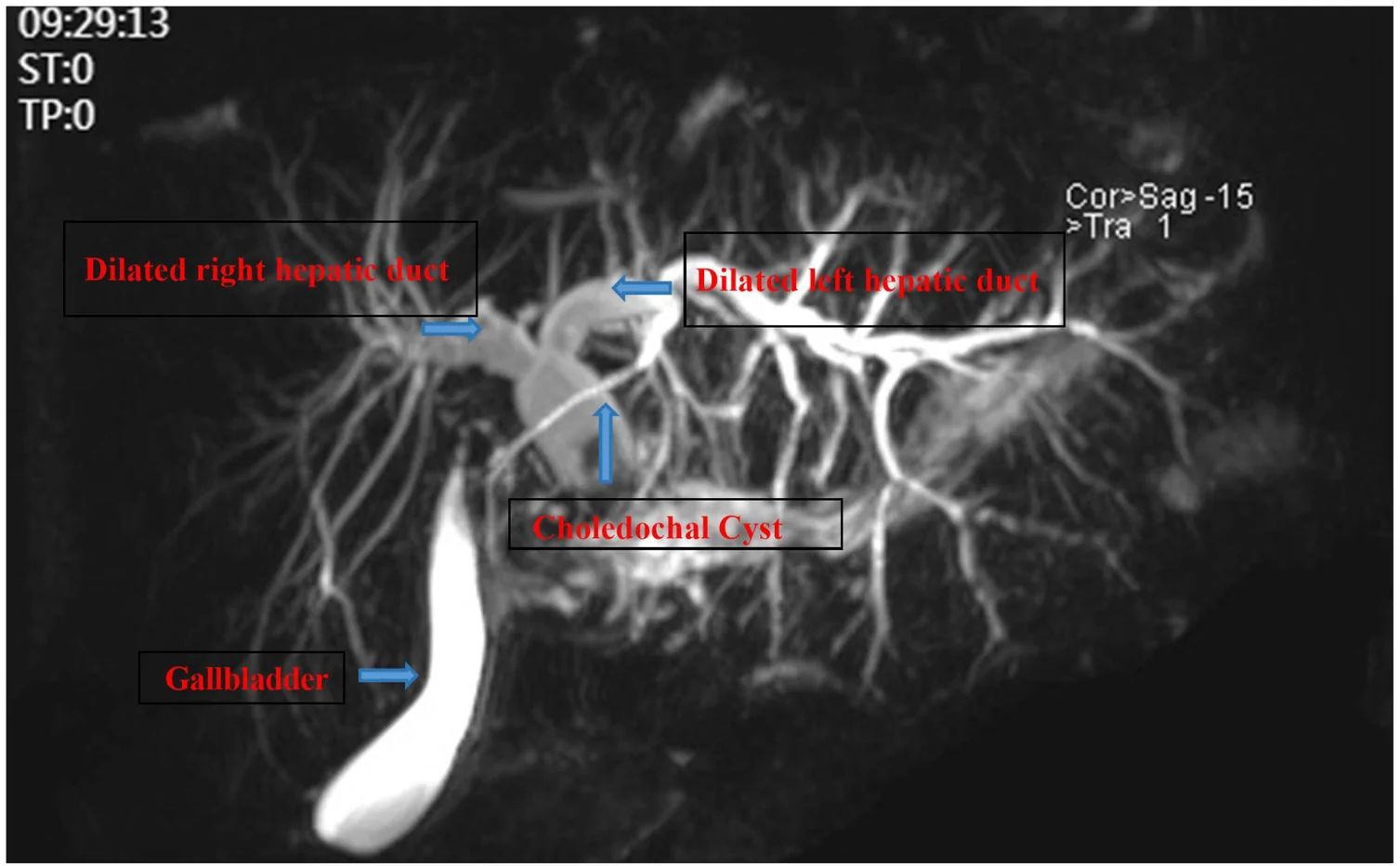
Comparative diagram of combined diagnosis of complex Todani type IVa choledochal cyst using US and MRCP. Comparative imaging of abdominal ultrasound (US) and magnetic resonance cholangiopancreatography (MRCP) in a patient with complex Todani type IVa choledochal cyst. Upper panel: MRCP 3D reconstruction demonstrates saccular dilatation of the common bile duct, common hepatic duct, and bilateral intrahepatic bile ducts, with marked dilatation of left and right hepatic ducts, and an intraluminal filling defect consistent with choledocholithiasis. Lower panel: Grayscale US identifies a cystic mass at the hepatic hilum corresponding to the dilated common bile duct cyst, with detectable intrahepatic biliary dilatation. Key anatomical structures are annotated with text labels and directional arrows.

**Figure 3:**
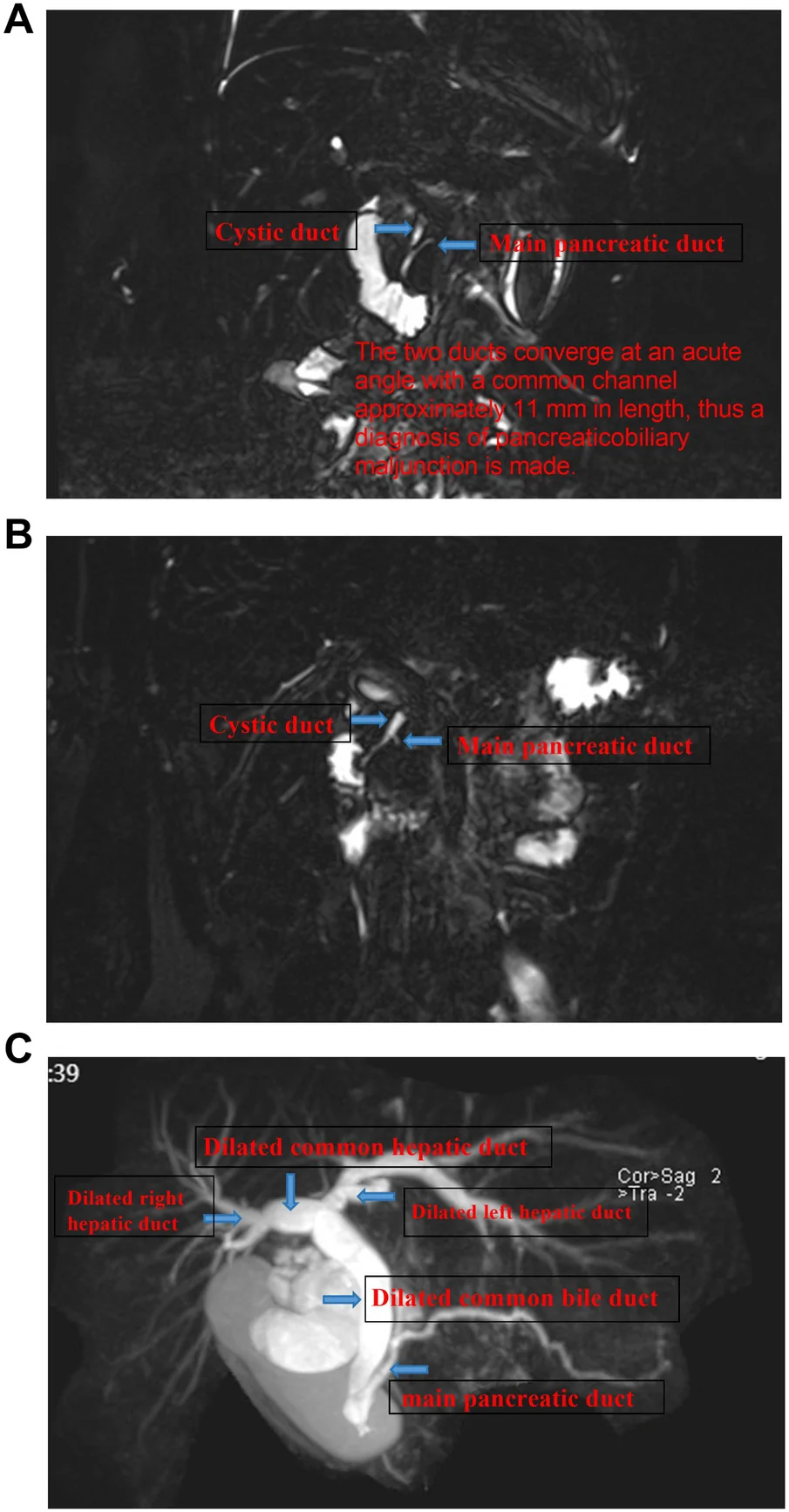
Three sets of typical illustrative images of pancreaticobiliary maljunction (PBM) on MRCP. Typical MRCP manifestations of pancreaticobiliary maljunction (PBM). **(A)** The common bile duct and main pancreatic duct converge at an acute angle outside the duodenal wall, forming a long common channel measuring 11.0 mm, accompanied by dilatation of the hepatic duct and left intrahepatic bile duct. **(B)** Elongated common channel (12 mm) formed by the proximal common bile duct and pancreatic duct, with gallbladder enlargement and tortuous cystic duct. **(C)** Diffuse dilatation of extrahepatic and intrahepatic bile ducts, with pancreaticobiliary confluence creating a 12-mm long common channel and concurrent choledocholithiasis. Arrows and text annotations mark the common bile duct, main pancreatic duct, and abnormal confluence segment.

**Figure 4:**
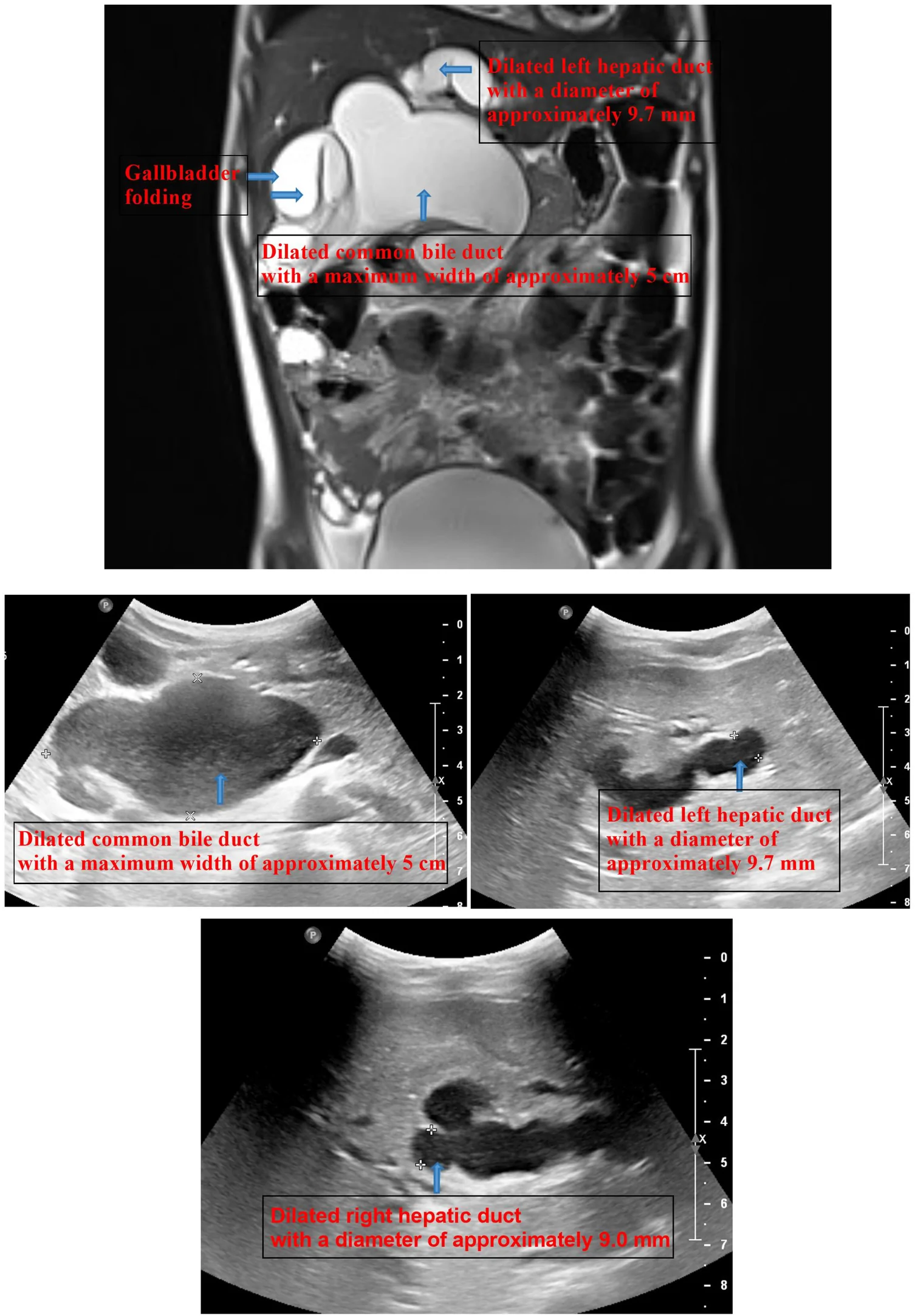
Severe extensive dilatation of intrahepatic bile ducts plus cystic biliary stasis/surrounding inflammatory changes (severe case of type IVa). Imaging findings of severe intrahepatic bile duct dilatation and biliary sludge deposition in advanced Todani type IVa choledochal cyst. Top row: Coronal MRCP images show massive cystic dilatation of the common bile duct (maximum diameter 5 cm), marked dilatation of bilateral intrahepatic bile ducts with a beaded appearance, gallbladder folding and tortuous cystic duct, and mild pancreatic duct dilatation. Bottom panels: Corresponding US scans confirm giant extrahepatic cystic dilatation and dilated left/right hepatic ducts; sonographic report demonstrates biliary sludge within the cyst and gallbladder inflammatory changes. Pericystic inflammatory infiltration and mild perihepatic free fluid are noted on MRI. All dilated biliary segments are annotated with arrows and dimension labels.

Figure 2 shows the imaging comparison of US and MRCP for typical Todani IVa choledochal cyst; Figure 3 shows the typical MRCP manifestations of pancreaticobiliary maljunction (PBM); Figure 4 shows imaging features of severe pericystic inflammation combined with extensive intrahepatic bile duct dilatation. All key lesion areas are annotated with arrows for intuitive display.

### 3.3 Correlation between preoperative radiological features and surgical selection

Individualized surgical procedures were performed based on cyst type, anatomical variations and disease severity. The distribution of procedures and their association with core radiological features are shown in Table 3. Of 104 patients, cyst resection + Roux-en-Y hepaticojejunostomy was the dominant procedure (85 cases, 81.7%), followed by hepatic portoplasty (11 cases, 10.6%) mainly for hilar involvement and common hepatic duct stenosis, hepatectomy (5 cases, 4.8%) for type V cysts with localized intrahepatic dilatation and liver injury, and combined individualized procedures (3 cases, 2.9%) for complex anatomical malformations with multifocal disease (Table 3).

**Table 3:** Correlation between preoperative radiological features and surgical selection.

Radiological feature	Surgical procedure [No. of cases (proportion/%)]	*χ*^2^ value	*P* value
	Cyst resection + Roux-en-Y hepaticojejunostomy	Hepatic portoplasty	Hepatectomy
Cyst diameter			
≤5 cm	72 (84.7)	4 (36.4)	0 (0.0)
>5 cm	13 (15.3)	7 (63.6)	5 (100.0)
Concomitant PBM			
Yes	41 (48.2)	9 (81.8)	4 (80.0)
No	44 (51.8)	2 (18.2)	1 (20.0)
Intrahepatic bile duct dilatation			
Extensive dilatation	22 (25.9)	8 (72.7)	4 (80.0)
No/mild dilatation	63 (74.1)	3 (27.3)	1 (20.0)

Correlation analysis showed that preoperative radiological features including cyst diameter >5 cm, concomitant PBM, extensive intrahepatic bile duct dilatation and severe pericystic inflammation/adhesion were significantly associated with the selection of complex procedures (hepatic portoplasty, hepatectomy, combined surgery) (*P* < 0.05). In contrast, patients with cyst diameter ≤5 cm and no intrahepatic involvement mostly underwent standard cyst resection + Roux-en-Y anastomosis, with lower surgical complexity and trauma.

### 3.4 Analysis of postoperative complications and risk factors

The follow-up period ranged from 3 to 24 months (median: 12.5 months). All prognostic outcome analyses were based on valid follow-up data ≥6 months. Overall, 17 cases (16.3%) developed early postoperative complications: biliary fistula (5, 29.4%), anastomotic stenosis (4, 23.5%), cholangitis (5, 29.4%) and pancreatitis (3, 17.6%). mid-term liver function abnormalities occurred in 9 cases (8.7%). No perioperative deaths or severe adverse events were recorded (Table 4).

**Table 4:** Analysis of postoperative complications and risk factors.

Risk factor	*β* value	SE value	OR value	95%CI	*P* value
Incomplete preoperative radiological assessment	1.448	0.412	4.26	2.03–8.94	<0.001
Todani type IVa/V cyst	1.125	0.376	3.08	1.47–6.42	0.003
Concomitant pancreaticobiliary maljunction (PBM)	1.357	0.394	3.89	1.81–8.36	<0.001
Radiological evidence of cyst wall necrosis	0.986	0.408	2.68	1.21–5.94	0.015
Constant	−2.754	0.523	0.064	–	<0.001

OR, odds ratio; 95%CI, 95% confidence interval.

*P* < 0.05 indicates statistical significance.

Univariate analysis was used to screen risk factors for early postoperative complications, followed by multivariate Logistic regression (Table 4). Incomplete preoperative radiological assessment, Todani type IVa/V cysts, concomitant PBM and radiological evidence of cyst wall necrosis were identified as independent risk factors (*P* < 0.05). Patients with incomplete preoperative radiological assessment had a 4.26-fold higher risk of early complications, while those with PBM had a 3.89-fold higher risk, highlighting the critical role of accurate preoperative radiological evaluation in mitigating postoperative risks.

### 3.5 Diagnostic performance of the combined radiological predictive model

**Figure 5:**
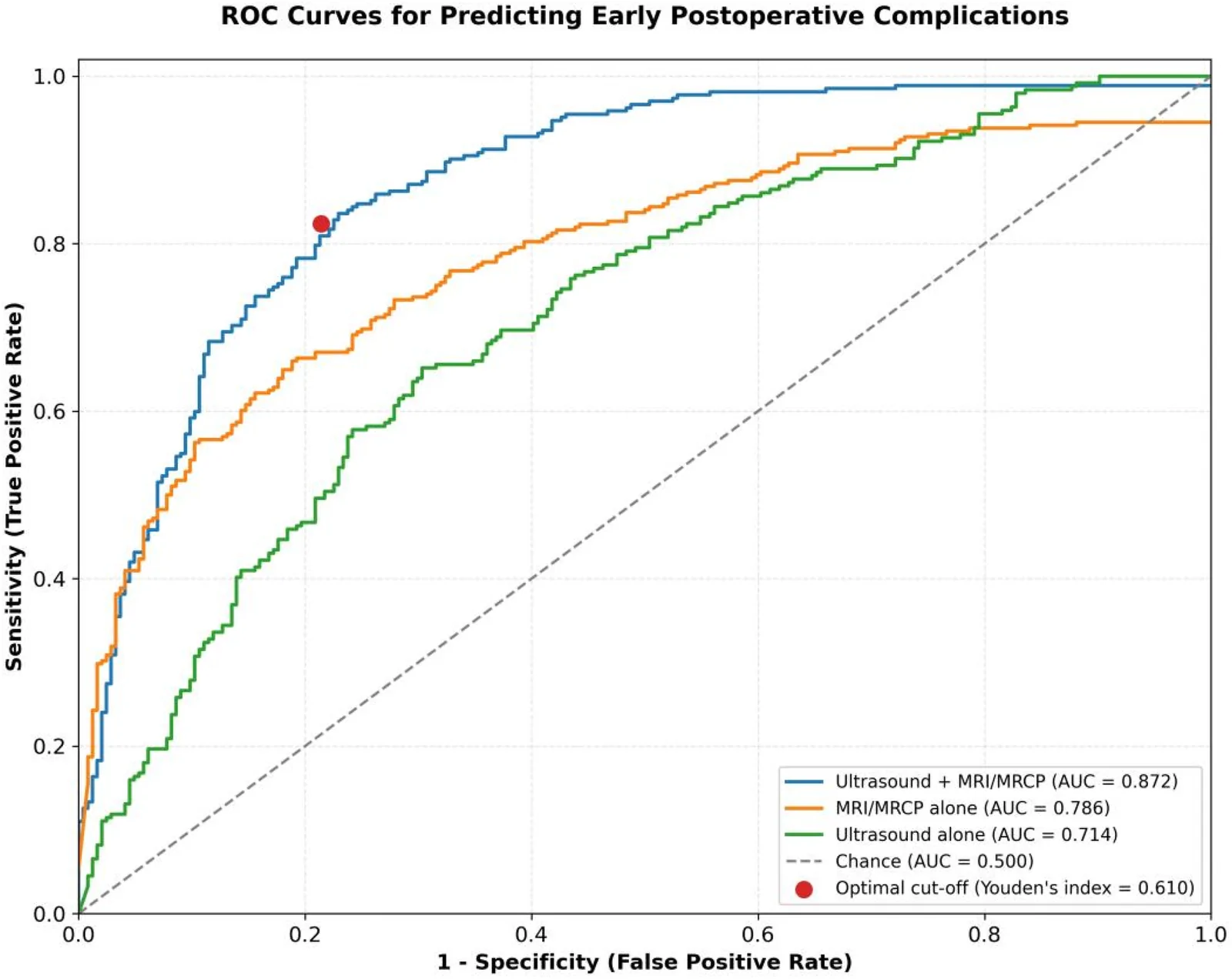
ROC curves of different imaging modalities for predicting early postoperative complications in pediatric CCC.

A combined predictive model for early postoperative complications was constructed using three core radiological parameters: cyst diameter (US), PBM and intrahepatic bile duct dilatation (MRI/MRCP), and ROC curves were plotted to evaluate predictive performance (Figure 5). The combined US and MRI/MRCP model yielded an AUC of 0.872 (95%CI: 0.795–0.949), with a maximum Youden index of 0.610. At the optimal cut-off value, the sensitivity was 82.4%, specificity 78.6%, positive predictive value 72.3% and negative predictive value 87.5%, demonstrating excellent overall predictive performance for identifying high-risk patients.

The predictive performance of single modalities was inferior to the combined approach: MRI/MRCP alone had an AUC of 0.786, and US alone 0.714, further confirming that combined US and MRI/MRCP is the optimal preoperative imaging strategy for pediatric CCC, balancing screening accuracy and prognostic value (Table 5).

**Table 5:** Diagnostic performance of different imaging modalities for predicting early postoperative complications in pediatric CCC.

Imaging modality	AUC (95%CI)	Sensitivity (%)	Specificity (%)	Positive predictive value (%)	Negative predictive value (%)	Youden index
Ultrasound	0.714	71.2	68.5	60.1	78.2	0.397
MRI/MRCP	0.786	77.6	75.3	67.4	83.6	0.529
Ultrasound + MRI/MRCP	0.872 (0.795–0.949)	82.4	78.6	72.3	87.5	0.610

### 3.6 Analysis of perioperative surgical indicators

Perioperative indicators were compared across surgical groups to explore the impact of preoperative radiological assessment on surgical trauma and recovery efficiency (Table 6). The standard cyst resection + Roux-en-Y anastomosis group had significantly shorter operative time, lower intraoperative blood loss and shorter postoperative hospital stay compared to the complex procedure groups (hepatic portoplasty, hepatectomy, combined surgery) (*P* < 0.05).

**Table 6:** Analysis of perioperative surgical indicators.

Surgical procedure	No. of cases	Operative time (min)	Intraoperative blood loss (ml)	Postoperative hospital stay (d)	Reoperation cases (%)
Cyst resection + Roux-en-Y hepaticojejunostomy	85	125.6 ± 22.4	18.7 ± 5.2	7.2 ± 1.5	1 (1.2)
Hepatic portoplasty	11	189.3 ± 31.6	32.5 ± 8.7	10.5 ± 2.3	1 (9.1)
Hepatectomy	5	217.5 ± 40.2	45.8 ± 11.3	13.1 ± 3.0	1 (20.0)
Combined surgery	3	242.0 ± 45.8	52.6 ± 13.5	14.7 ± 3.5	0 (0.0)
Statistic	–	*F* = 27.643	*F* = 19.826	*F* = 32.197	*χ*^2^ = 8.924
*P* value	–	<0.001	<0.001	<0.001	0.030

Stratified analysis further showed that patients with complete preoperative radiological assessment (US + MRI/MRCP) had a median operative time shortened by approximately 35 min, a 21.3% reduction in intraoperative blood loss, and a 1.8-day shorter postoperative hospital stay compared to those with incomplete assessment, indicating that precise preoperative radiological planning can simplify surgical procedures, reduce trauma and accelerate postoperative recovery. Only 3 patients (2.9%) required reoperation for anastomotic stenosis and recurrent cholangitis, with a low reoperation rate; all reoperated patients had incomplete preoperative radiological assessment and complex anatomical malformations.

### 3.7 Comparison of baseline characteristics between good and poor prognosis groups

Patients were divided into a good prognosis group (no liver function abnormalities or recurrent biliary complications, *n* = 95) and a poor prognosis group (liver function abnormalities/recurrent mid-term complications, *n* = 9) based on 12-month postoperative liver function and imaging findings. Baseline characteristics were compared (Table 7).

**Table 7:** Comparison of baseline characteristics between good and poor prognosis groups.

Indicator	Good prognosis group (*n* = 95)	Poor prognosis group (*n* = 9)	Statistic	*P* value
Gender [*n* (%)]			*χ*^2^ = 0.021	0.885
Male	39 (41.1)	3 (33.3)	–	–
Female	56 (58.9)	6 (66.7)	–	–
Age [years, median (range)]	3.1 (2 months–12 years)	3.5 (6 months–10 years)	*Z* = 0.317	0.751
Todani classification [*n* (%)]			*χ*^2^ = 9.362	0.002
Type I/III	75 (78.9)	3 (33.3)	–	–
Type IVa/V	20 (21.1)	6 (66.7)	–	–
Preoperative imaging modality [*n* (%)]			*χ*^2^ = 0.104	0.747
US + MRI/MRCP	90 (94.7)	8 (88.9)	–	–
Other combinations	5 (5.3)	1 (11.1)	–	–

Data are presented as *n* (%). Poor prognosis is defined as liver function abnormalities, recurrent biliary stenosis or recurrent cholangitis at 12 months postoperatively.

No significant differences were observed in gender, age distribution or preoperative imaging modality between the two groups (*P* > 0.05), indicating balanced baseline comparability. In terms of Todani classification, the proportion of type IVa/V cysts was significantly higher in the poor prognosis group (66.7% vs. 21.1%, *χ*^2^ = 9.362, *P* = 0.002), suggesting that cyst type may be associated with poor mid-term prognosis. The small sample size of the poor prognosis group limits the generalizability of mid-term outcome findings, requiring further validation in larger cohorts.

### 3.8 Stratified analysis of mid-term prognosis

Stratified analysis based on preoperative radiological features is shown in Table 8. The overall good prognosis rate was 91.3% (95/104), and the poor prognosis rate was 8.7% (9/104). All patients with poor prognosis had preoperative radiological high-risk features, including Todani type IVa/V cysts, concomitant PBM, extensive intrahepatic bile duct dilatation and severe pericystic inflammation. Patients with ≥2 radiological high-risk factors had a 5.72-fold higher risk of poor mid-term prognosis compared to those with no high-risk factors, indicating that preoperative radiological high-risk features can serve as predictors of mid-term outcomes, warranting intensified postoperative follow-up and intervention.

**Table 8:** Stratified analysis of mid-term prognosis.

Preoperative radiological high-risk factor	Good prognosis group (*n* = 95)	Poor prognosis group (*n* = 9)	*χ*^2^ value	*P* value
Todani type IVa/V cyst	20 (21.1)	6 (66.7)	9.362	0.002
Concomitant PBM	43 (45.3)	8 (88.9)	6.815	0.009
Extensive intrahepatic bile duct dilatation	24 (25.3)	7 (77.8)	10.748	<0.001
Severe pericystic inflammation and adhesion	31 (32.6)	6 (66.7)	4.217	0.040
≥2 high-risk factors	17 (17.9)	7 (77.8)	13.290	<0.001

## 4 Discussion

Pediatric CCC is a heterogeneous group of congenital biliary malformations, where accurate preoperative assessment, individualized surgical planning and postoperative risk stratification are key to improving prognosis (1, 19). This retrospective analysis of 104 children systematically investigated the core role of preoperative radiological assessment in surgical decision-making, complication prediction and mid-term outcomes.

### 4.1 Diagnostic value of different preoperative imaging modalities

Preoperative radiological assessment is the “navigational cornerstone” of CCC management, requiring not only detection of the cyst but also precise Todani classification, clear delineation of the pancreaticobiliary junction, evaluation of intrahepatic bile duct involvement and perilesional adhesions to provide comprehensive anatomical data for surgical decision-making (1, 20).

This study confirms that MRI/MRCP, with its non-invasive water imaging, multiplanar reconstruction and high soft-tissue resolution, represents the gold standard for preoperative classification and anatomical evaluation of CCC, achieving a Todani classification accuracy of 96.2%, with detection rates of 92.3% for PBM and 95.7% for intrahepatic bile duct dilatation, significantly superior to US and conventional CT. These findings align with recent international studies: Dillman et al. demonstrated that MRCP can clearly visualize the fine anatomy of the pancreaticobiliary junction without contrast agents, enabling precise assessment of the extent, severity and branch involvement of intrahepatic bile duct dilatation, which is critical for differentiating complex type IVa/V cysts and avoiding intraoperative anatomical misjudgment (21, 22); Wang et al. further reported that MRCP achieves nearly 98% classification accuracy for complex CCC, directly visualizing the relationship between the cyst and the portal vein/hepatic artery, thereby reducing the risk of intraoperative vascular injury (23, 24). MRCP's unique ability to overcome interference from intestinal gas and adipose tissue, and to fully reconstruct the biliary tree, makes it irreplaceable in preoperative planning.

US is the first-line screening tool for pediatric CCC, offering unique advantages of non-invasiveness, no radiation, portability, bedside availability, dynamic monitoring and cost-effectiveness, making it ideal for primary screening, outpatient evaluation and short-term postoperative follow-up (14). In this study, US achieved a classification accuracy of 82.7%, effectively detecting common bile duct dilatation, cyst size and initial intrahepatic involvement, consistent with findings by Miron et al. (25–27). However, US has notable limitations: imaging quality is affected by intestinal gas, abdominal fat and patient cooperation, leading to suboptimal visualization of the pancreaticobiliary junction and distal biliary obstruction; evaluation of complex cysts and diffuse intrahepatic dilatation is subjective, often failing to fully meet the requirements of complex surgical planning (14, 28). Thus, US alone cannot serve as the basis for definitive diagnosis or surgical planning, requiring combination with MRCP for comprehensive evaluation.

CT is an important complementary modality in preoperative CCC assessment, not a routine first-line examination. Its core advantage lies in evaluating the relationship between the cyst and surrounding tissues, and detecting liver parenchymal lesions, helping surgeons preoperatively predict dissection difficulty and plan surgical access (16). However, CT exposes children to ionizing radiation, a particular concern in this vulnerable population (29), so it is only recommended for complex cases with unresolved anatomical doubts after US and MRCP, or suspected severe pericystic infection/perforation, with strict adherence to indications.

Synthesizing the advantages and limitations of each modality, the combination of US for initial screening and MRI/MRCP for precise anatomical evaluation represents the optimal preoperative strategy for pediatric CCC, balancing diagnostic performance, safety and cost-effectiveness, enabling rapid screening and accurate classification to lay a solid foundation for subsequent surgical treatment.

### 4.2 Guidance of radiological features on individualized surgical strategies

The core goals of surgical treatment for pediatric CCC are complete resection of the diseased cyst, relief of biliary obstruction, correction of PBM, reconstruction of patent biliary drainage, and ultimately prevention of mid-term complications such as recurrent cholangitis, pancreatitis, cirrhosis and malignant transformation (30–33). Due to wide variations in cyst type, size, anatomy and comorbidities, a single procedure cannot be applied to all cases, and surgical planning is highly dependent on preoperative anatomical information provided by imaging. Accurate radiological stratification is a prerequisite for individualized, minimally invasive and precise surgical care.

This study identified cyst diameter >5 cm, concomitant PBM, extensive intrahepatic bile duct dilatation and severe pericystic inflammation as core radiological indicators of increased surgical complexity, significantly associated with the selection of complex procedures. Todani classification is the central determinant of surgical strategy, with distinct pathological features dictating resection extent and reconstruction methods. Type I/III cysts accounted for 65.4% of cases, with lesions confined to the distal common bile duct or common hepatic duct, no extensive intrahepatic involvement, and relatively simple PBM anatomy. The majority of these patients were treated with standard complete cyst resection + Roux-en-Y hepaticojejunostomy, the internationally recognized first-line procedure for type I/III CCC (34–36). This standardized approach achieves complete cyst resection and reliable biliary drainage, with a low rate of early postoperative complications.

In contrast, type IVa/V cysts represent complex CCC, involving both extrahepatic and intrahepatic bile ducts with diffuse or segmental dilatation, often complicated by intrahepatic stenosis and segmental atrophy. Simple extrahepatic cyst resection cannot fully resolve intrahepatic obstruction or cholestasis (37). In this study, 80% of type IVa/V cysts required complex procedures, including hepatic portoplasty, partial hepatectomy and combined cyst resection with intrahepatic ductoplasty, consistent with mid-term follow-up studies of complex CCC by Cazares et al. (35, 38). These complex procedures demand advanced surgical skills, requiring precise MRCP localization of intrahepatic involvement and strictures to plan appropriate resection and reconstruction, avoiding residual lesions or inadequate drainage.

Beyond classification, other radiological parameters directly impact surgical difficulty and strategy selection: large cyst diameter (>5 cm) causes compression of surrounding structures, increasing dissection complexity (39, 40); PBM exacerbates pancreatic reflux risk, requiring meticulous dissection and management of the pancreaticobiliary junction intraoperatively (32, 41); extensive intrahepatic dilatation indicates severe cholestasis, necessitating enhanced postoperative drainage management (42); severe pericystic inflammation leads to dense adhesions and obscured anatomy, significantly increasing the risk of intraoperative bleeding and adjacent organ injury (39). These findings confirm that radiological parameters can objectively quantify surgical complexity, enabling preoperative risk stratification: standard procedures for low-risk patients to minimize operative time, and advanced preoperative preparation and contingency planning for high-risk patients, facilitating precise and individualized surgical decision-making.

### 4.3 Predictive value of radiological parameters for postoperative complications and mid-term prognosis

Preoperative radiological assessment not only guides surgical planning but also predicts perioperative outcomes and mid-term quality of life. Extraction of key radiological risk factors and construction of predictive models enable early identification of high-risk patients and targeted intervention, optimizing the entire management pathway of CCC (43, 44).

Core innovative advantages of this study compared with previous literature: Different from previous studies that only focused on the single diagnostic efficacy of imaging modalities, this study further clarified the internal mechanism of standardized preoperative radiological assessment improving perioperative and mid-term prognostic outcomes from three key dimensions. First, precise MRCP anatomical localization accurately defines cyst boundary, PBM fusion type and intrahepatic lesion scope, avoids blind intraoperative exploration, and effectively shortens operative time. Second, preoperative identification of high-risk features such as pericystic severe adhesion and vascular compression enables targeted intraoperative protection, avoids unexpected biliary and vascular injuries, and reduces intraoperative blood loss and surgical trauma. Third, mid-termradiological risk stratification guides individualized surgical scheme selection, matches surgical complexity with disease severity, avoids insufficient resection or excessive surgical intervention, simplifies surgical procedures, and significantly optimizes perioperative recovery indicators and prognosis. In addition, the self-constructed US-MRCP combined prediction model in this study has higher predictive efficacy than single imaging modality, which can accurately identify high-risk children with adverse postoperative outcomes and provide targeted clinical intervention basis.

Multivariate Logistic regression identified incomplete preoperative radiological assessment, complex type IVa/V cysts, concomitant PBM and cyst wall necrosis as independent risk factors for early postoperative complications (including biliary fistula, pancreatic fistula, abdominal infection, intestinal obstruction and pancreatitis). Incomplete assessment, often due to omission of MRCP, leads to intraoperative errors such as cyst remnant or inappropriate biliary reconstruction, directly triggering postoperative complications; type IVa/V cysts are associated with extensive intrahepatic disease, greater surgical trauma and higher drainage difficulty, elevating complication risk; PBM causes persistent pancreatic reflux, leading to recurrent postoperative pancreatitis and cholangitis; cyst wall necrosis indicates severe local inflammation, increasing the risk of anastomotic healing failure. Kang et al. reported that CCC patients without MRCP assessment have higher postoperative complication rates, corroborating the necessity of complete preoperative radiological evaluation (45); Nguyen et al. demonstrated that type IVa cysts have higher complication rates than type I cysts, identifying complex classification as a core driver of adverse outcomes (46); Li et al. confirmed that PBM is a risk factor for postoperative pancreatitis and cholangitis recurrence, consistent with our findings, emphasizing the need for intensified postoperative acid and enzyme suppression in these patients (47).

In terms of prognostic predictive performance, this study innovatively constructed a combined US and MRI/MRCP radiological model, achieving an AUC of 0.872, sensitivity of 82.4% and specificity of 78.6%, significantly outperforming single-modality imaging. This model can rapidly identify high-risk patients for early postoperative complications, guiding preemptive interventions such as enhanced perioperative antimicrobial therapy, optimized surgical technique and prolonged drainage, effectively reducing complication risk.

Mid-term prognostic analysis further revealed that patients with ≥2 preoperative radiological high-risk factors had a 5.72-fold higher risk of poor mid-term outcomes (including biliary stenosis recurrence, recurrent cholangitis, cirrhosis and malignant transformation), demonstrating that preoperative radiological features can serve as stratification markers for mid-term prognosis. For these high-risk patients, clinical management must extend beyond successful surgery to establish a mid-term standardized follow-up protocol, with regular US and MRCP to monitor biliary drainage, liver function and cyst recurrence, enabling timely intervention for mid-term complications and maximizing mid-term quality of life.

In summary, preoperative radiological assessment is integral to the entire management of pediatric CCC, playing an irreplaceable role from initial screening and classification to surgical planning, complication prediction and mid-term follow-up. Clinical practice should promote the standardized US + MRI/MRCP evaluation protocol, refine risk stratification based on radiological features, and implement individualized management strategies to achieve early diagnosis, precise assessment, optimal treatment and mid-term follow-up, comprehensively improving overall outcomes for children with CCC.

### 4.4 Study limitations and future perspectives

This is a single-center retrospective study, with potential selection bias in gender distribution and case enrollment; the limited sample size and relatively short mid-term follow-up duration (median 12.5 months) preclude subgroup analysis of minimally invasive surgery, and the impact of radiological features on mid-term cholangiocarcinoma risk requires extended follow-up and multi-center validation. Future research could explore the application of AI-assisted radiological classification in preoperative assessment.

## 5 Conclusion

Preoperative comprehensive radiological assessment is a core component in the surgical management of pediatric choledochal cysts, enabling precise individualized surgical planning and effective prediction of early postoperative complication risk. The combination of US and MRI/MRCP represents the optimal preoperative imaging strategy, clearly delineating cyst type and pancreaticobiliary anatomical variations. Strengthening standardized preoperative radiological assessment, early identification of high-risk factors and implementation of targeted surgical management can significantly reduce the incidence of early postoperative complications and improve mid-term outcomes in children with choledochal cysts.

## Data Availability

The original contributions presented in the study are included in the article/Supplementary Material, further inquiries can be directed to the corresponding author.
